# The Ectodomain of TLR3 Receptor Is Required for Its Plasma Membrane Translocation

**DOI:** 10.1371/journal.pone.0092391

**Published:** 2014-03-20

**Authors:** Jelka Pohar, Nina Pirher, Mojca Benčina, Mateja Manček-Keber, Roman Jerala

**Affiliations:** 1 National Institute of Chemistry, Hajdrihova 19, Ljubljana, Slovenia; 2 EN-FIST Centre of Excellence, Ljubljana, Slovenia; 3 Faculty of Chemistry and Chemical Technology, University of Ljubljana, Ljubljana, Slovenia; University of Tennessee Health Science Center, United States of America

## Abstract

Toll-like receptor 3 (TLR3) is a dsRNA sensing receptor that is localized in the cellular compartments but also at the plasma membrane. Overexpression of UNC93B1 promoted localization of TLR3, but not other nucleic acid sensing TLRs, to the plasma membrane. Here we show that UNC93B1 itself is localized at the plasma membrane. We investigated the role of different domains of TLR3 on cell signaling by preparing chimeric receptors between TLR3 and TLR9 where each of the transmembrane segments or cytosolic domains has been exchanged. While the ectodomain completely governs ligand specificity and the cytosolic TIR domain determines the engagement of the signaling adapters as well as the potentiation of receptor activation by UNC93B1, the ectodomain but not transmembrane segment or cytosolic domain determines plasma membrane localization of TLR3. Nevertheless, TLR3 receptor and ligand endocytosis as well as endosomal acidification are important for the robust signaling of TLR3.

## Introduction

Toll-like receptors (TLRs) are a family of pattern recognition receptors which promote efficient innate immune response by recognizing the conserved pathogen associated molecular patterns (PAMPs) or endogenous danger associated signals (DAMPs) [1], [2]. Recognition of those signals and subsequent activation of signaling pathways lead to cytokine and chemokine production and maturation of adaptive immune response [3]. TLRs have common domain structure with a large N-terminal horseshoe-shaped ectodomain which consists of conserved leucine-rich repeats (LRRs), a transmembrane helix and a C-terminal cytoplasmic Toll-interleukin-1 receptor (TIR) domain [1]. TLRs differ in their ligand specificity, cellular localization of the receptors and signaling pathways [3]. TLR1, TLR2, TLR4, and TLR6 are expressed at the plasma membrane and recognize PAMPs in the bacterial cell wall components from Gram-positive and Gram-negative bacteria, yeast and fungi. TLR5 is also localized at the plasma membrane and recognize flagellin. TLR3 and TLR9 are members of nucleic-acid sensing TLRs along with TLR7, TLR8 and TLR13. They are mainly confined to intracellular compartments [1], [4]. Upon stimulation by agonists they translocate from endoplasmic reticulum (ER) to the endosomes where they can encounter the internalized nucleic acid ligands [5], [6]. Ligands of nucleic acid-sensing (NAS) TLRs are viral or bacterial nucleic acids [1], [7]. TLR3 recognizes double-stranded RNA (dsRNA) which is formed during the replication process of many viruses [8], [9] and TLR9 recognizes the nonmethylated CpG motives in microbial DNA [10]. Activation of TLR3 and binding affinity with ligand are pH and length dependent. The strongest response to dsRNA is achieved between pH 5.7 and 6.7 [11] which corresponds to endosomal pH range [12].

Localization, trafficking and therefore also signaling of NAS TLRs to endosomes depends on the accessory protein – Unc93b1 (mouse Unc93b1) [13], [14]. Unc93b1 is a 12-helical-membrane spanning ER resident protein [15]. It interacts with transmembrane segments (TM segment) of TLR3, TLR7, TLR9, TLR11 and TLR13 [16]–[18]. Mice carrying the Unc93b1 3D mutation (H412R) are highly susceptible to infection with intracellular pathogens (*e.g.* mouse cytomegalovirus, *Listeria monocytogenes* and *Staphylococcus aureus*) and have impaired response via TLR3, TLR7 and TLR9 [15]. Report on the interaction between the N-terminal cytoplasmic tail of Unc93b1 and the cytoplasmic domain of intracellular TLRs demonstrated the role of Unc93b1 in TLR trafficking. This region on Unc93b1, especially the residue D34, regulates the association and interaction affinity for nucleic-acid sensing TLRs. D34A mutation markedly weakens interaction with TLR9 and promotes interaction with TLR7 [19].

Upregulation of UNC93B1 (human UNC93B1) increased the amount of TLR3, but not TLR7, TLR8 or TLR9, at the plasma membrane [20]. In our study we aimed to identify the domain harboring the plasma membrane localization signal of TLR3. This was achieved through chimeric TLR3-TLR9 receptors where the transmembrane, cytosolic or ectodomains have been exchanged. Rather than in the transmembrane or cytosolic domain as reported previously for other NAS TLRs, we found that the ectodomain of TLR3 governs the plasma membrane localization. Moreover, we showed that the TLR3 at the plasma membrane colocalizes with poly(I:C). However, potent TLR3 activation requires the endosomal localization since inhibitors of endosomal acidification and endocytosis greatly reduced the response of cells with the endogenous levels of UNC93B1 as well as in cells overexpressing UNC93B1.

## Materials and Methods

### Cell cultures

Human embryonic kidney cells (HEK) 293 and HEK293T were cultivated in DMEM (Invitrogen) supplemented with 10% (v/v) FBS (Gibco) at 37°C in 5% CO_2_.

### Plasmids and reagents

Expression plasmids containing sequences of TLR3 (pUNO-hTLR3), TLR9 (pUNO-hTLR9-HA), and UNC93B1 (pUNO1-hUNC93B1) were from InvivoGen, TLR3-mCer (pcDNA3-hTLR3-mCerulean) containing plasmid was prepared in our lab [9], TLR9-YFP (pcDNA3-hTLR9-YFP) was from Addgene, plasmid constitutively expressing *Renilla* luciferase–phRL-TK was from Promega, pmCherry-C1 was from Clontech Laboratories. The following plasmids were gifts: plasmid coding for firefly luciferase under NF-κB promoter (pELAM-1-luciferase) was gift from C. Kirschning (Institute for Medical Microbiology, University of Duisburg-Essen, Essen, Germany), plasmid coding for firefly luciferase under IFN-β promoter (pIFN-β–luciferase; from J. Hiscott, Departments of Microbiology and Medicine, McGill University, Montreal, QC, Canada), Unc93b1-GFP and EEA1-Tomato were gift from T. Espevik (Norwegian University of Science and Technology, Trondheim, Norway) and pmCerulean-C1 was provided by D. Piston (Vanderbilt University, Nashville, TN, USA).

Cells were treated with different TLR ligands: polyinosinic-polycytidylic acid - poly(I:C) (InvivoGen) and type B CpG-oligodeoxynucleotide ODN10104 (Coley Pharmaceutical Group). Cells were treated with different inhibitors: inhibitor of actin polymerization - cytochalasin D (Sigma Aldrich), inhibitor of dynamin – Dynasore (Sigma Aldrich) and inhibitor of endosomal acidification - bafilomycin A (LC Laboratories).

### DNA constructs preparation

All chimeric DNA constructs were created by means of a PCR overlap extension technique and cloned into pUNO vector (InvivoGen). Proofreading DNA polymerase AccuPrime Pfx (Invitrogen) was used in all reactions. TLR3-9-3 stands for a construct where TM segment of TLR3 has been exchanged with TM segment of TLR9. TLR9-3-9 stands for a construct where TM segment of TLR9 has been exchanged with TM segment of TLR3. TLR3-3-9 stands for a construct where cytoplasmic domain of TLR3 has been exchanged with cytoplasmic domain of TLR9. TLR9-9-3 stands for a construct where cytoplasmic domain of TLR9 has been exchanged with cytoplasmic domain of TLR3. All constructs were sequenced. Primer sequences are available upon request. Fusions with fluorescent protein to the C-terminus of the chimeric DNA constructs were also prepared. Constructs were cloned into pcDNA3 vector. TLR3-9-3 was cloned between *Cla*I and *Bam*HI restriction sites into native pcDNA3-hTLR3-mCerulean. TLR9-3-9, TLR3-9-9 and TLR9-9-3 were cloned between *Bsp*EI and *Bam*HI restriction sites into pcDNA3-hTLR9-YFP. Resulting constructs were TLR3-9-3-mCerulean, TLR9-3-9-YFP, TLR3-3-9-YFP and TLR9-9-3-YFP. A list of all constructs with their amino acid composition is summarized Table 1.

**Table 1:** Constructs used in experiments and their amino acid composition.

Construct	Vector	Amino acid composition	Designation
wt hTLR3	pUNO		TLR3
wt hTLR3-mCer	pcDNA3		TLR3-mCer
wt hTLR9-HA	pUNO		TLR9
wt hTLR9-YFP	pcDNA3		TLR9-YFP
TLR3 with tmTLR9	pUNO	TLR3 (M1-L704) TLR9 (C818-L838) TLR3 (E726-H904)	TLR3-9-3
TLR3 with tmTLR9 - mCer	pcDNA3		TLR3-9-3 - mCer
TLR9 with tmTLR3 - HA	pUNO	TLR9 (M1-D817) TLR3 (F705-F725) TLR9 (C839-E1032)	TLR9-3-9
TLR9 with tmTLR3 - YFP	pcDNA3		TLR9-3-9 - YFP
TLR3 with cytoTLR9-HA	pUNO	TLR3 (M1- F725) TLR9 (C839-E1032)	TLR3-3-9
TLR3 with cytoTLR9-YFP	pcDNA3		TLR3-3-9 - YFP
TLR9 with cytoTLR3-HA	pUNO	TLR9 (M1- L838) TLR3 (E726-H904)	TLR9-9-3
TLR9 with cytoTLR3-YFP	pcDNA3		TLR9-9-3 - YFP

HA-hemagglutinin tag.

mCer – mCerulean fluorescent protein.

YFP – yellow fluorescent protein.

tm – transmembrane segment.

cyto – cytosolic domain.

pUNO-UNC93B1-mCherry-Myc and pUNO-Unc93b1-mCherry-Myc were created by means of a PCR overlap extension technique and cloned into pUNO vector (InvivoGen). UNC93B1, Unc93b1 and mCherry were cloned from pUNO1-hUNC93B1, Unc93b1-GFP and pmCherry-C1 plasmids respectively. Myc tag was embedded within primers used in PCR reaction.

### Transfection and reporter gene assay

HEK293 cells were plated onto CoStar White 96-well plates (Corning) at 2.2×10^4^ cells/well. After 24 h, the cells were transfected with a following plasmids: pIFN-β-luciferase (40 ng DNA/well) or ELAM1-luciferase reporter plasmid (40 ng DNA/well), pUNO-hTLR3-HA (20 ng DNA/well), pUNO-hTLR9-HA (20 ng DNA/well) or TLR3-TLR9 chimeric constructs (20 ng DNA/well), pUNO1-hUNC93B1 (1 ng DNA/well) and phRL-TK (5 ng DNA/well). Empty vector pcDNA3 (20 ng DNA/well) was used as a negative control. Plasmids were transfected using Lipofectamine 2000 reagent according to manufacturer's instructions (Invitrogen). 24 h post transfection, the cells were stimulated with TLR ligands: poly(I:C) (10 μg/ml) and ODN10104 (10 μg/ml). 18 h after treatment, the cells were lysed in Passive Lysis Buffer (Promega). The expression of the firefly and *Renilla* luciferase reporter gene was analyzed using the dual luciferase assay. Luminescence was quantified using the plate reader OrionII (Berthold Technologies). The relative luciferase expression (relative luciferase unit - RLU) for each sample was calculated by normalizing firefly luciferase activity for constitutive *Renilla* luciferase activity measured within the same sample.

### SDS-PAGE and western analysis

HEK293T cells were seeded onto 12-well plates (Techno Plastic Products) at 2.2×10^5^ cells/well. After 24 h, at 50% confluence, they were transfected with TLR3, TLR9, TLR3-TLR9 chimeric constructs and control vector pcDNA3 (900 ng DNA/well). 48 h after transfection, the cells were lysed using RIPA buffer (50 mM Tris pH 7.5, 150 mM NaCl, 1% (v/v) Triton X-100, 0.1% SDS, 0.5% DOC) with Complete Mini protease inhibitors (Roche), sonicated and centrifuged. The protein-containing supernatants were harvested and the total protein amount was quantified using the BCA protein assay (Sigma-Aldrich). The cell extracts (30 μg of total proteins) were incubated at 65°C for 5 min in sample buffer (SDS with 2-mercaptoethanol) and loaded onto a 12% SDS-PAGE gel. After electrophoresis, proteins were transferred onto nitrocellulose membranes Hybond-ECL (GE Healthcare) and detected with following primary antibodies: mouse monoclonal anti-TLR3 (IMG-315A, Imgenex), rabbit anti-HA (H6908, Sigma-Aldrich), mouse anti-β-actin (3700, Cell Signaling). Used secondary antibodies were: goat anti-mouse IgG-HRP (sc-2005, Santa Cruz) and goat anti-rabbit IgG-HRP (ab6721, Abcam). The blots were developed using SuperSignal West Pico Chemiluminescent Substrate (Pierce). Membranes were recorded with G:BOX Chemi using GeneSnap software (Syngene).

### Confocal microscopy

HEK293T cells were seeded onto eight-well tissue culture chambers (Ibidi) at 2.2×10^5^ cells/well. After 24 h, the cells were transfected with 150 ng DNA/well of the TLR3-mCer, TLR9-YFP or TLR3-TLR9 chimeric constructs fused with mCerulean or YFP, UNC93B1 (30 ng DNA/well), UNC93B1-mCherry-Myc (30 ng DNA/well) or Unc93b1-mCherry-Myc (30 ng DNA/well). For detection of early endosomes the cells were cotransfected with EEA1-Tomato (30 ng DNA/well). Localization was visualized 48 h post transfection. A plasma membrane was stained with either SynaptoRed (Biotioum), Cholera Toxin Subunit B Alexa Fluor 555 or Cholera Toxin Subunit B Alexa Fluor 647 (Molecular Probes, Invitrogen). Lysosomes were stained with LysoTracker Red DND-99 or LysoTracker Green DND-26, endosomes were stained with Transferrin AlexaFluor 633 conjugate and ER was stained with ER-Tracker Red or ER-Tracker Blue-White DPX (all from Molecular Probes, Invitrogen). Poly(I:C) (LMW) labeled with rhodamine was from InvivoGen. Cell expressing endogenous levels of UNC93B were stimulated for 4 h with 10 μg/ml of labeled poly(I:C). Cell overexpressing UNC93B were stimulated for 1 h with 10 μg/ml of labeled poly(I:C). Images were acquired using the Leica TCS SP5 inverted laser-scanning microscope on a Leica DMI 6000 CS module equipped with a HCX Plane-Apochromat lambda blue 63× oil-immersion objective with NA 1.4 (Leica Microsystems). Colocalization was identified with LAS AF software. Fluorescence intensity from two channels - TLR and plasma membrane were measured in line profile (n = 9) in distance of 3 μm crossing membrane. Florescence intensities per distance were plotted (GraphPad Prism software) and maximum fluorescence intensities of TLR were compared with those of plasma membrane.

### Statistical Analysis

Error bars represent mean S.D. of triplicate samples. Data were compared for significance using the one-tailed unpaired Student t-test and were considered significant with p value of 0.05.

## Results

### TLR3 and UNC93B1 colocalize at the plasma membrane

TLR3 has been found at the cell surface in several cell types such as human lung fibroblast cell line MRC-5 [21] and some epithelial and endothelial cells (human corneal epithelial cells, human lung microvascular endothelial cell, human umbilical vein endothelial cells) [23]–[25]. As we described before, in contrast to TLR9, UNC93B1 directs TLR3 to the plasma membrane in addition to endosomes [20]. We wanted to establish the location of UNC93B1 with respect to TLR3 and TLR9.

**Figure 1:**
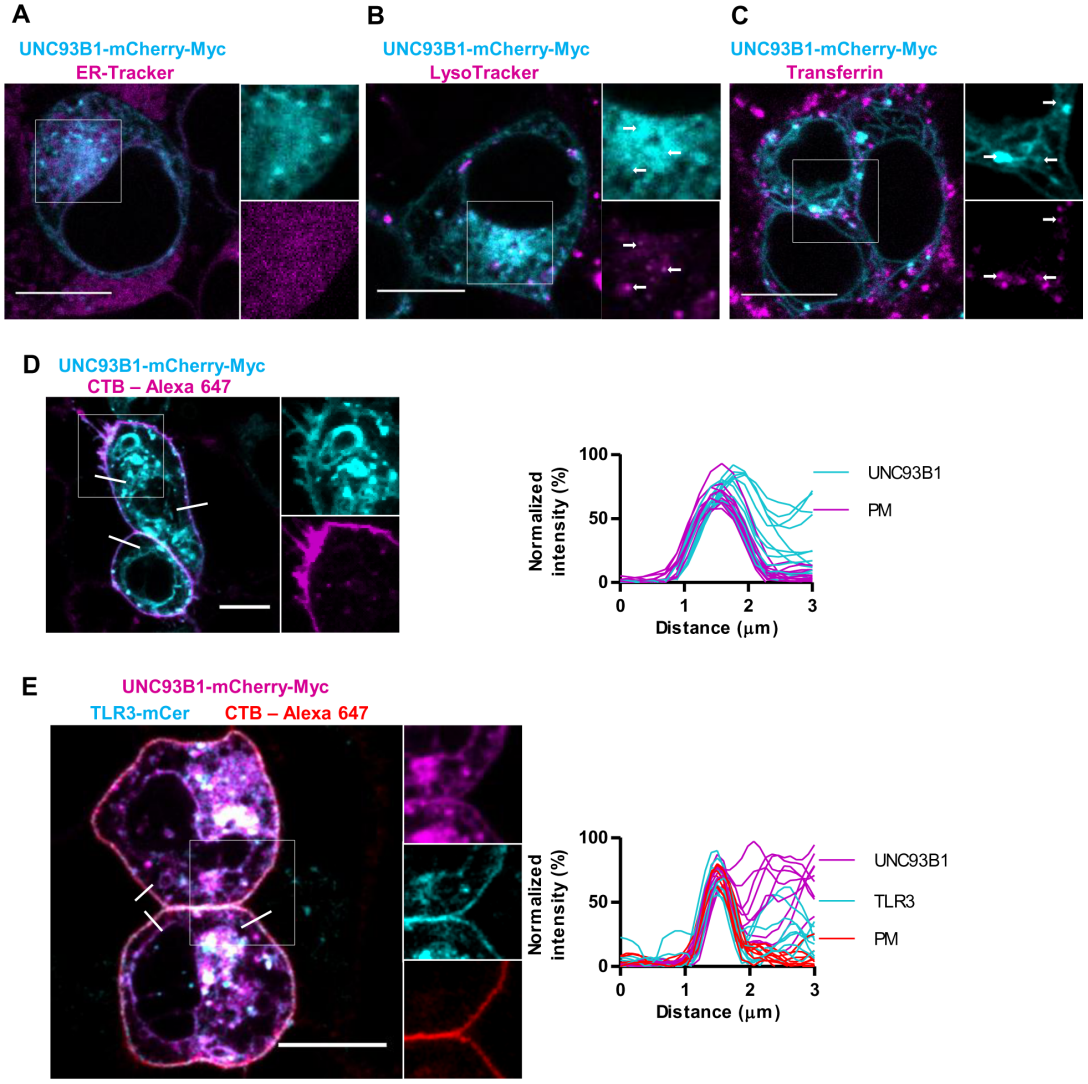
UNC93B1 is localized in the ER and in the lysosomes. (A–D) HEK293T cells were transfected with a UNC93B1-mCherry-Myc (cyan). (A) ER was dyed with ER-Tracker Blue-White DPX. (B) Lysosomes were marked with LysoTracker Green DND-26. (C) Endosomes were stained with Transferrin AlexaFluor 633 conjugate. (D) Plasma membrane was dyed with CTB-Alexa 647. All dyes are shown in magenta. White arrows indicate colocalization. (E) HEK293T cells were transfected with a UNC93B1-mCherry-Myc (magenta) and TLR3-mCer (cyan). Plasma membrane was dyed with CTB-Alexa 647 (red). Membrane localization was evaluated from plots of normalized fluorescence intensities of UNC93B1-mCherry-Myc and TLR3-mCer and plasma membrane (PM) within 3 μm line profiles (n = 9). Three representative lines are marked on merged images. Images are selected from three independent experiments. Scale bars, 10 μm.

Accordingly to Kim *et al.* and Fukui *et al.* Unc93b1 is mainly expressed in the ER and endosomes [13], [19]. Since TLR3 localizes to the cell surface upon UNC93B1 overexpression we tested whether UNC93B1 can reach the plasma membrane. HEK293T cells were transfected with UNC93B1-mCherry-Myc and dyed with specific markers for endoplasmic reticulum (ER), lysosomes, endosomes and plasma membrane. We found that UNC93B1 is mainly localized in the ER (Fig. 1A) and lysosomes (Fig. 1B) but less common in the transferrin containing early endosomes (Fig. 1C). UNC93B1-mCherry-Myc was observed at the plasma membrane in cells not expressing TLR3 as well as in cell overexpressing TLR3 (Fig. 1D and Fig 1E). Overexpression of both mouse and human UNC93B1 increased the protein levels of TLR3 (Fig. S1 C and D). However, mouse Unc93b1 does not reach the plasma membrane and it does not promote translocation of human TLR3 towards the plasma membrane (Fig. S1 A and B). In contrast to human UNC93B1 (Fig. S1C), mouse Unc93b1 does not translocate the differentially glycosylated TLR3 to the plasma membrane (Fig. S1D). The UNC93B1-mCherry-Myc and Unc93b1-mCherry-Myc both augmented signaling (Fig. S1E) and increased protein expression of TLR3 (Fig. S1D). This has been previously shown to be a consequence of the increased lifetime of TLR proteins associated with UNC93B1 [20].

### Poly(I:C) colocalizes with TLR3 at the cell surface

**Figure 2:**
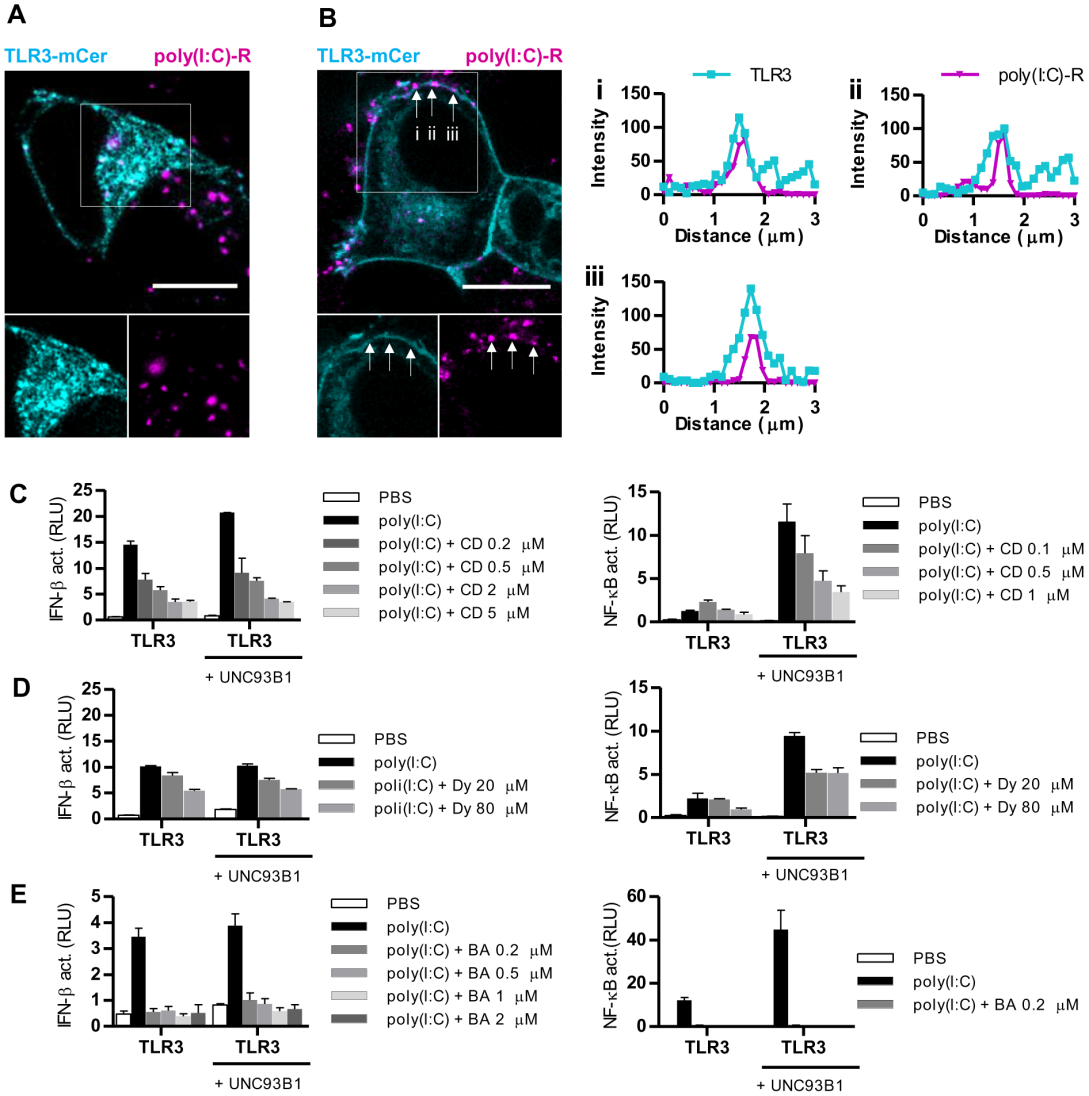
Poly(I:C) and TLR3 colocalize at the surface of plasma membrane. HEK293T cells were transfected with TLR3-mCer alone (A) or cotransfected with UNC93B1 (B). Cells were stimulated with rhodamine labeled poly(I:C) (poly(I:C)-R). (B) TLR3 and poly(I:C)-R colocalization was evaluated from plots (right) of fluorescence intensities within 3 μm line profiles (n = 3; i, ii, iii). Three representative speckles where cross-sections were analyzed are marked with the white arrows on the merged image. (C–E) HEK293 cells were transfected with TLR3 alone or with UNC93B1 encoding plasmid. Cells were cotransfected with IFN-β (left) or NF-κB (right) promoter reporter plasmid and *Renilla* reporter plasmid. Cells were simultaneously treated with poly(I:C) (10 μg/ml) and inhibitors. Cells were treated with increasing amounts (0.2–5 μM) of cytochalasin D (abbr. CD) (C), Dynasore (abbr. Dy) (20–80 μM) (D) or bafilomycin A (abbr. BA) (0.2–2 μM) (E). 8 h after treatment luciferase activity (RLU) was measured in the cell lysates. The results are represented by mean values with SD from triplicate wells. The representative data from three experiments are shown.

In order to investigate whether TLR3 and dsRNA colocalize at the cell surface, we stimulated cells with rhodamine-labeled poly(I:C). In cells that produced the endogenous amount of UNC93B1, poly(I:C) is mainly localized in the intracellular compartments (Fig. 2A). Similar intracellular localization of poly(I:C) was observed in cells with TLR3 at the cell surface. In addition, we observed colocalization of poly(I:C) with TLR3 at the plasma membrane (Fig. 2B left). We analyzed three regions (i, ii, iii in Fig. 2B) where this colocalization was observed. Fluorescence intensities of two channels – TLR3 and poly(I:C) overlap (Fig. 2B right).

However, it is not clear whether the signaling is initiated at the plasma membrane or TLR3 and dsRNA have to be internalized to trigger signaling. We demonstrated previously that UNC93B1 enhanced activation of TLR3 [20]. Inhibitors of endocytosis (cytochalasin D and Dynasore) [26], [27] (Fig. 2C and 2D) as well as endosomal acidification inhibitor (bafilomycin A) [27] (Fig. 2E), prevented activation of IFN-β (left) and NF-κB (right) pathway. We observed inhibition of TLR3 signaling in cells with the endogenous level of UNC93B1 as well as in cells overexpressing UNC93B1. Our data suggest that both acidic pH and endocytosis are important for the robust TLR3 response despite its surface localization where those inhibitors should not have any effect on signaling.

### Modularity of ligand recognition and signaling pathway selection of TLR3:TLR9 chimeras

**Figure 3:**
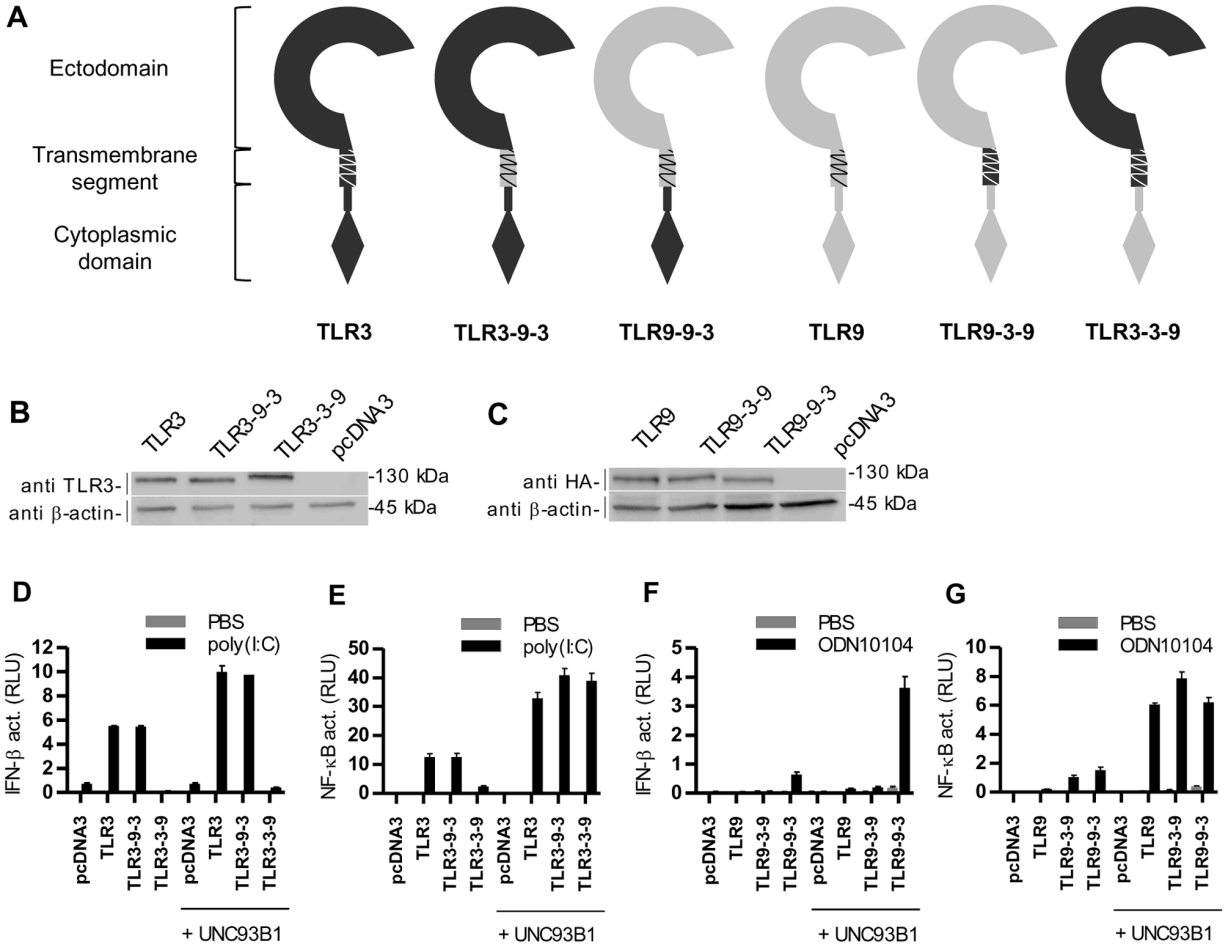
Response of chimeric constructs between TLR3 and TLR9. (A) Schematic representation of chimeric constructs where the transmembrane segments or cytoplasmic domains of human TLR3 and TLR9 receptors have been exchanged. (B, C) HEK293T cells were transfected with TLR3, TLR3-9-3 and TLR3-3-9 (B) or TLR9, TLR9-3-9 and TLR9-9-3 (C). Western blot was performed using anti-TLR3 or anti-HA antibodies. Anti–β-actin antibodies were used as a loading control. The representative data from three experiments are shown. (D-E) HEK293 cells were transiently transfected with TLR3, TLR9 or chimeric constructs TLR3-9-3, TLR3-3-9, TLR9-3-9, and TLR9-9-3 alone or with UNC93B1. Cells were transfected with IFN-β (D, F) or NF-κB (E, G) promoter reporter plasmids and *Renilla* reporter plasmid. After 18 h of stimulation with poly(I:C) (10 μg/ml) (D, E) or ODN10104 (10 μg/ml) (F, G), luciferase activity (RLU) was measured in the cell lysates. The results are represented by mean values with SD from triplicate wells. The representative data from three experiments are shown.

Molecular motifs determining endosomal translocation of NAS TLRs have been mapped to either the TM segment or to the cytoplasmic domain [28], [29]. A cytosolic juxtamembrane domain has been reported as essential for the endosomal localization of mouse and human TLR3 [30], [31]. A TM segment is required for the mouse TLR9 endosomal localization while the cytoplasmic domain has been reported to affect human TLR9 localization [32]–[34]. Unc93c1 physically interacts with TM segments of TLR3, TLR7, TLR8, TLR9 and TLR13 [16]. To determine which segments of TLR3 are important for the difference in cellular localization between TLR3 and TLR9, we prepared chimeric constructs, where we combined the ectodomains, TM segments and cytosolic domains of TLR3 and TLR9 in different combinations (Fig. 3A and Table 1; detailed description of chimeric receptors is provided in the section Materials and methods).

Expression levels of chimeric proteins were comparable (Fig. 3B and 3C). Agonistic ligand specificity was completely determined by respective TLR ectodomain. Chimeric proteins comprising ectodomain of TLR3 conferred response to poly(I:C) (Fig. 3D and 3E) and chimeric proteins comprising ectodomain of TLR9 responded to ODNs (Fig. 3F and 3G).

The signaling pathway that was activated upon stimulation was governed by the cytoplasmic domain of each chimeric receptor. The presence of the cytoplasmic domain of TLR3 induced activation of IFN-β as well as NF-κB response, while the constructs comprising the cytoplasmic domain of TLR9 activated NF-κB (Fig. 3E and 3G) but poorly activated the IFN-β pathway (Fig. 3D and 3F). This demonstrates that TIR domains independently determine the signaling pathway [28].

UNC93B1 augmented signaling of TLR3 and TLR9 as well as in all chimeric receptors (Fig. 3D-G). With respect to the enhancement of activation by the upregulation of UNC93B1 the effect was governed by the cytosolic TIR domain, while the TM segment had no effect suggesting that the affinity of the TM segment of TLR3 and TLR9 to the UNC93B1 is comparable. Response of constructs with the cytoplasmic domain of TLR9 was enhanced by up to 28 fold while UNC93B1 amplified the response of constructs with TLR3 cytoplasmic domain from 2 to 6 fold. Fold induction is calculated as the ratio of the response after UNC93B1 overexpression to the response without UNC93B1 overexpression (Table 2).

**Table 2:** Responsiveness and fold induction after UNC93B1 overexpression of constructs containing TLR3 and TLR9 domains.

	TLR3	TLR3-9-3	TLR9-9-3	TLR9	TLR9-3-9	TLR3-3-9
IFN-β response	**+**	**+**	**+**	***-***	***-***	***-***
Fold induction with UNC93B1	2	2	6	***/***	***/***	***/***
NF-κB response	**+**	**+**	**+**	**+**	**+**	**+**
Fold induction with UNC93B1	3	3	4	28	8	16

### The ectodomain of TLR3 is responsible for the UNC93B1-dependent translocation to the plasma membrane

**Figure 4:**
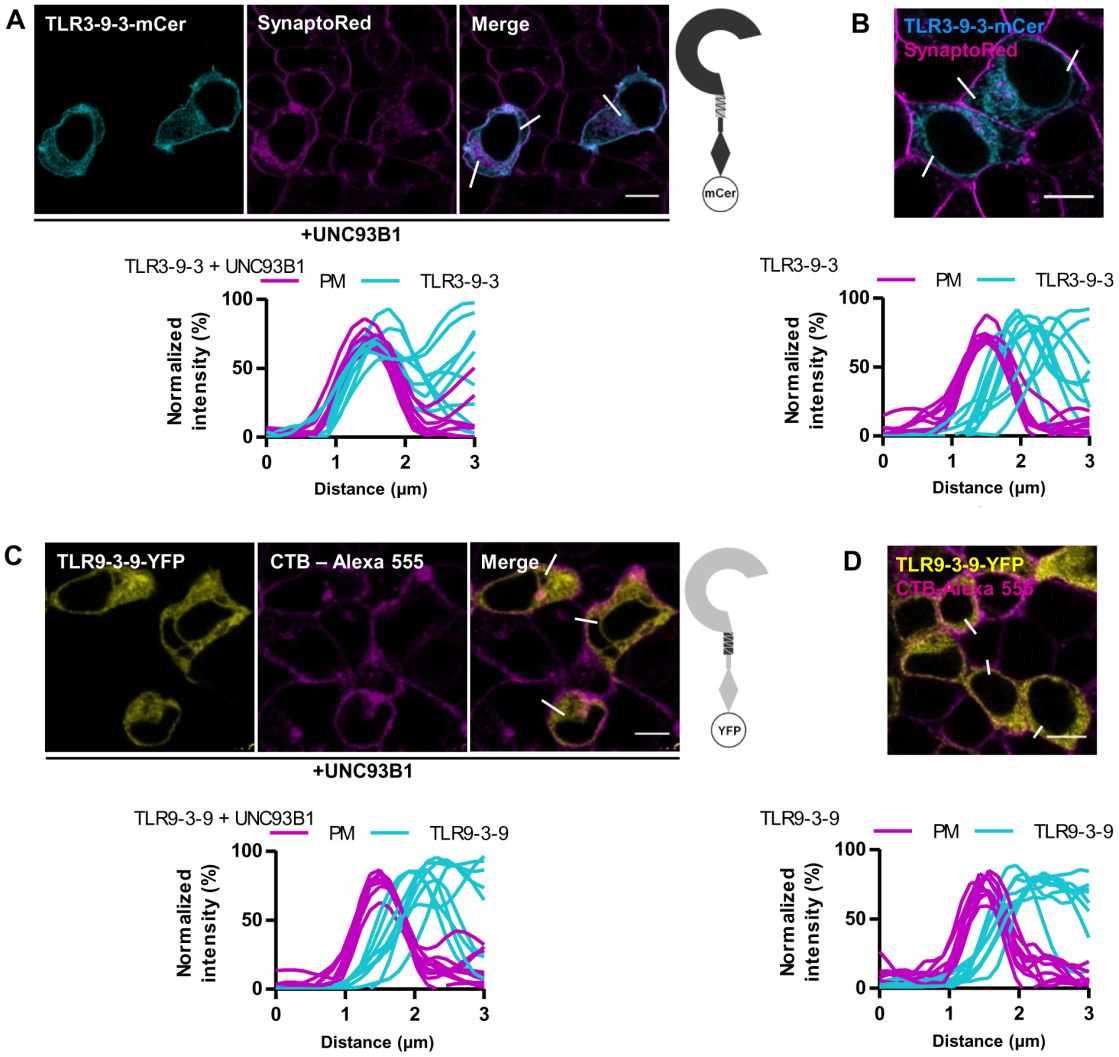
Localization of chimeric receptors with exchanged TM segments of TLR3 and TLR9. HEK293T cells were transiently transfected TLR3-9-3-mCer (A and B - cyan), TLR9-3-9-YFP (C and D - yellow), and with UNC93B1. Plasma membrane markers SynaptoRed and CTB-Alexa 555 are shown in magenta. (A) Localization of TLR3-9-3-mCer on plasma membrane in cells overexpressing UNC93B1. (B) Intracellular localization of TLR3-9-3-mCer in HEK293T without overexpression of UNC93B1. (C) Intracellular localization of TLR9-3-9-YFP in cells overexpressing UNC93B1. (D) Intracellular localization of TLR9-3-9-YFP in HEK293T without overexpression of UNC93B1. (A-D) Data are representative of three experiments. TLR membrane localization was evaluated from plots (bottom) of normalized fluorescence intensities of TLR and plasma membrane (PM) within 3 μm line profiles (n = 9). Three representative lines are marked on merged images. Images are selected from three independent experiments. Scale bars, 10 μm.

**Figure 5:**
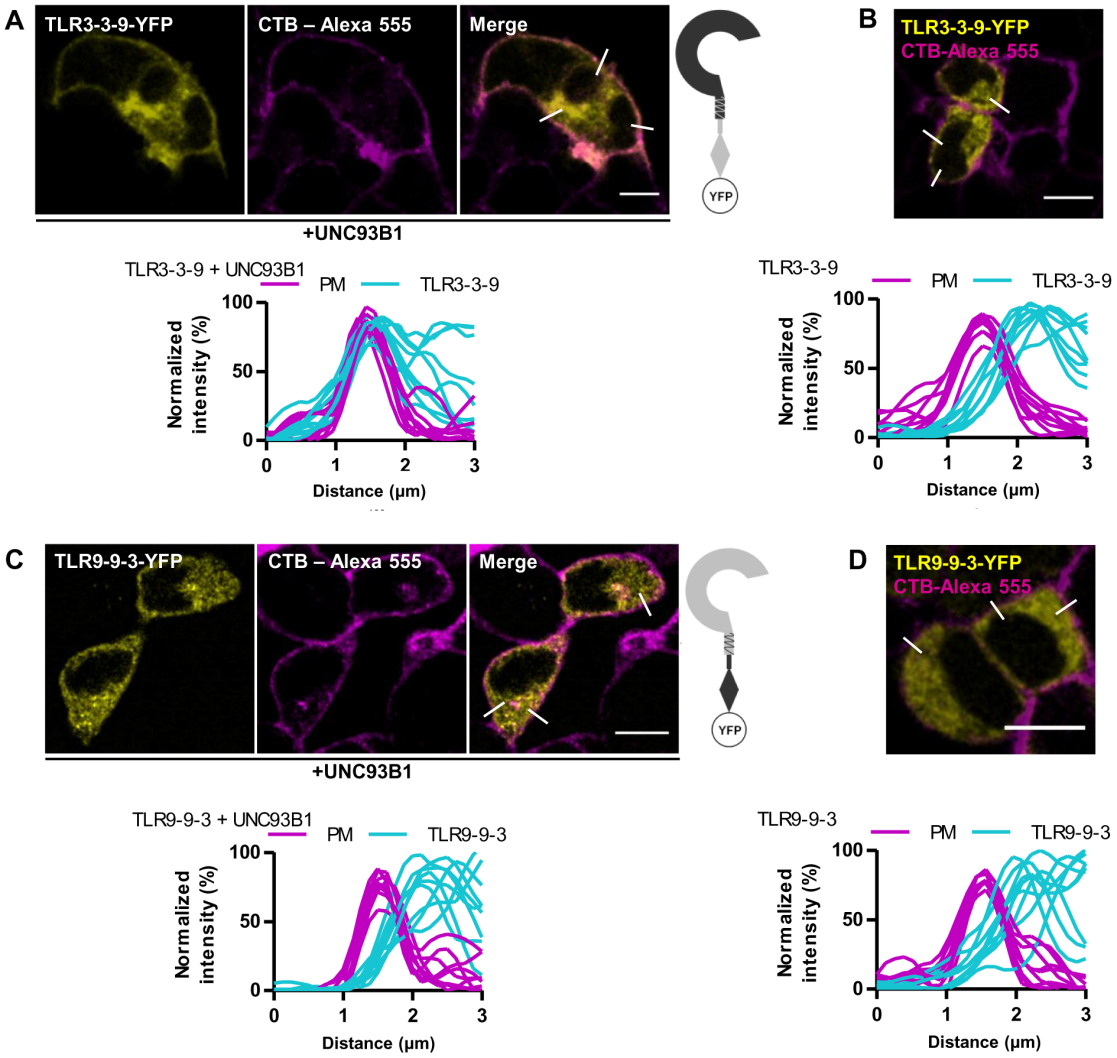
Localization of chimeric receptors with exchanged cytosolic domains of TLR3 and TLR9. HEK293T cells were transiently transfected TLR3-3-9-YFP (A and B - yellow) or TLR9-9-3-YFP (C and D - yellow) and with UNC93B1. Plasma membrane markers SynaptoRed and CTB-Alexa 555 are shown in magenta. (A) Localization of TLR3-3-9-YFP on plasma membrane in cells overexpressing UNC93B1. (B) Intracellular localization of TLR3-3-9-YFP in HEK293T without overexpression of UNC93B1. (C) Intracellular localization of TLR9-9-3-YFP in cells overexpressing UNC93B1. (D) Intracellular localization of TLR9-9-3-YFP in HEK293T without overexpression of UNC93B1. (A–D) Data are representative of three experiments. TLR membrane localization was evaluated from plots (bottom) of normalized fluorescence intensities of TLR and plasma membrane (PM) within 3 μm line profiles (n = 9). Three representative lines are marked on merged images. Images are selected from three independent experiments. Scale bars, 10 μm.

Chimeric TLRs tagged with a C-terminal fluorescent protein were used to detect the cellular localization of chimeric proteins. TLR3-9-3-mCer chimera localized at the cell surface in case of UNC93B1 overexpression (Fig. 4A) but TLR9-3-9-YFP was restricted to intracellular compartments (Fig. 4C), suggesting that neither the TM segment nor the cytosolic domain are the decisive factor directing the localization of TLR3 towards the plasma membrane. UNC93B1 delivered chimeric protein TLR3-3-9-YFP to the plasma membrane (Fig. 5A), but TLR9-9-3-YFP (Fig. 5C) exhibited only intracellular localization. In cells expressing low amount of UNC93B1 all receptors localized like the wild type TLR3 and TLR9 (Fig. 4B and 4D, Fig. 5B and 5D). Analysis of colocalization confirmed that in cells overexpressing UNC93B1 the profiles from TLR3-9-3-mCer and TLR3-3-9-YFP overlap with the profiles of the plasma membrane marker (Fig. 4A bottom, Fig. 5A bottom) similar as in the wtTLR3 [20] while traces from TLR9-3-9-YFP or TLR9-9-3-YFP and plasma membrane demonstrate no membrane location (Fig. 4C bottom, Fig. 5C bottom). These results suggest that the motif governing the UNC93B1-dependent localization of TLR3 to the plasma membrane resides in the ectodomain of TLR3.

**Figure 6:**
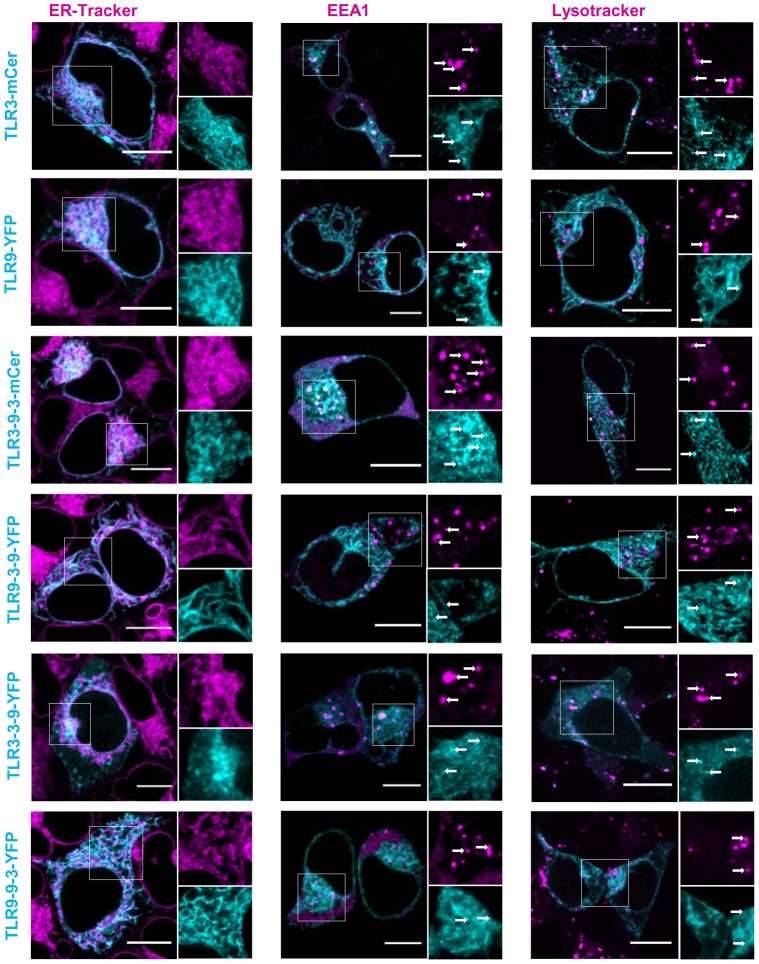
Structural alterations do not affect translocation of chimeric receptors from ER to endosomes and lysosomes. HEK293T cells were transiently transfected with TLR3-mCer, TLR9-YFP, TLR3-9-3-mCer, TLR9-3-9-YFP, TLR3-3-9-YFP or TLR9-9-3-YFP. ER was dyed with ER-Tracker Red, lysosomes were marked with LysoTracker Red DND-99. To stain endosomes, cells were cotransfected with EEA1-Tomato. All dyes are shown in magenta. White arrows indicate colocalization. Images are selected from three independent experiments. Scale bars, 10 μm.

TLR3 and TLR9 are mainly localized in the ER [5], [9] and so are the chimeric receptors (Fig. 6). However, structural alterations in chimeric receptors could prevent their exit from the ER. Despite those alternations all chimeric proteins are able to translocate to endosomes and lysosomes. All receptors are also normally responsive to their ligands (Fig. 3D–3E). If translocation to endosomes of NAS TLRs is compromised (for example 3d mutation in Unc93b1), there is no activation of immune response [34].

## Discussion

In addition to several cell lines that constitutively express TLR3 at the plasma membrane [22]–[25], cell surface localization of TLR3 is also induced by the viral infection. TLR3 was detected at the cell surface in airway epithelial cells A549 and human bronchial epithelial cells BEAS-2B upon the respiratory syncytial virus (RSV) and rhinovirus (RV) infection, respectively [35], [36].

We showed that TLR3 and poly(I:C) colocalize at the surface of the plasma membrane of UNC93B1 overexpressing cells. While some reports suggest that activation occurs entirely in acidic endosomes [37], several studies reported that surface expressed TLR3 participates in the recognition of dsRNA and triggers the signaling pathway. This was corroborated by the inhibition of signaling by anti-TLR3 antibodies [22], [35], [36]. Bouteiller et al. demonstrated that in contrast to dsRNA, activating antibodies against TLR3 ectodomain activated TLR3-CD32 chimeric protein from the cell surface in a pH-independent manner. Binding of endogenous extracellular dsRNA to TLR3 at cell membrane is still possible but due to higher pH probably less efficient than in endosomes [11]. Our results show that both, endocytosis and endosomal acidification, are important for the robust poly(I:C)-induced signaling through TLR3. Endosomal activation of TLR3 seems to be much stronger than activation of cell surface associated TLR3, possibly due to smaller number of receptors at the cell surface or cellular localization of TRIF adapter (TIR-domain-containing adapter-inducing interferon-β) [38]. TRIF adapter can reach the plasma membrane but only by the aid of a myristoylated TRAM adapter (TRIF-related adaptor molecule) [39]. Unlike TLR4, TLR3 does not recruit the TRAM adapter [40]. DsRNA is important for activation of TLR3 and cytosolic dsRNA sensors and enters into cells by clathrin dependent endocytosis [41]. However, it is not clear if TLR3 itself can facilitate the internalization of dsRNA, similar as the TLR4 which was rapidly endocytosed following the binding of its agonist, [42] since the TLR3 binds dsRNA weakly at the physiological pH. However, binding of membrane TLR3 to polymeric poly(I:C) to is likely to have high avidity. Poly(I:C) can be internalized with the aid of Raftlin which interacts with nucleic acids at the plasma membrane [43] or in a complex with the antimicrobial peptide LL-37 through FPRL-1 receptor [44].

We reported that UNC93B1 is responsible for trafficking of differentially glycosylated TLR3, but not TLR7, TLR8 or TLR9, to the plasma membrane [20]. However, in unstimulated cells UNC93B1 is localized in ER and in lysosomes, consistent with previous studies [13], [20]. Localization of TLR9 to the cell membrane has been reported in HEK293 as a consequence of stimulation with CpG DNA [5] on human peripheral blood mononuclear cells (PBMC) stimulated by LPS [45] and on mouse intestinal epithelial cells after exposure of cells to DNA from pathogenic *Salmonella enterica* [46]. Recently it has been reported that Unc93b1 is also required for the surface appearance of mouse TLR9 [47], however, we could not detect the surface localization of TLR9 on HEK293 cells. The reason for those differences may be that we investigated localization of the human and not mouse TLR9. On the other hand forced cell surface localization of TLR9 induced severe autoimmune defects in mice [48].

We found that receptors with the cytosolic domain of TLR9 were far more responsive to the UNC93B1 overexpression. These data further support the idea, that the cytosolic domain defines the strength of interaction between TLR9 and UNC93B1 in agreement with the effect of cytosolic D34A mutation of Unc93B1 which suppressed the selectivity of Unc93b1 for TLR9 vs. TLR7 [19]. Our results demonstrate that the motif directing the UNC93B1 dependent sorting of TLR3 to the plasma membrane most likely resides within the ectodomain of TLR3. Unc93b1 alone could interact with segments of TLR3 ectodomain [49], [50]. It is also possible that an additional accessory protein participates in sorting of TLR3 to the cell surface, versus sorting to the endosomes. We observed that mouse Unc93b1, similar as human UNC93B1, augment TLR3 signaling and increases TLR3 protein expression but it does not translocate human TLR3 towards the plasma membrane. TLR3 surface localization is also associated with appearance of differentially glycosylated TLR3 [20], [51] which does not appear in cells overexpressing mouse Unc93b1. This demonstrates that there could be a difference in interaction between mouse and human UNC93B1 with TLR3. So far, plasma membrane localization of TLR3 was discovered in human cell lines [21]–[24], [35], [36].

Recent study of Kim *et al.* reported that Unc93b1 interacts with ectodomains of NAS TLRs, which was ascribed to the juxtamembrane region of TLR3 and TLR9. Those residues were important for the proper translocation of receptors from ER to endosomes [50]. Those results supports our conclusion that UNC93B1 also interacts with the ectodomain segments of NAS TLRs and not only transmembrane and cytoplasmic domains as it was shown before [16], [19]. Those residues could be important for phenomena we discovered; however translocation of TLR3 has not been tested by Kim *et al.* Besides the mentioned acidic amino acid residues, there must be another motif in the ectodomain that governs different trafficking of TLR3 in comparison to other NAS TLRs. Nevertheless, these results support our conclusion and findings from Qi *et al.* [49] that UNC93B1 can interact with ectodomain segments of TLR3. Qi *et al*. reported that knockdown of UNC93B1 reduced full-length TLR3 at the plasma membrane. They defined two loops named Loop 1 and Loop 2 located in LLR12 and LRR20, respectively. Deletions in Loop 1 inhibit TLR3 ectodomain secretion, whereas changes in Loop 2 only decreased secretion [49]. The deletion of 64 amino acids (Δ64) between LRR10 and 12 is present in TLR3 isoform expressed in primary human astrocytes [52] and comprise Loop 1. Δ64 interacted with UNC93B1 weaker than the wild type TLR3. Residues in Loop 1 are also required for proper translocation of TLR3 to the endosomes [51]. Qi *et al.* also showed that the human TLR3 P554S polymorphism located in Loop 2 reduced the amount of TLR3 at the cell surface relative to the WT receptor [49]. This polymorphism is associated with increased susceptibility to herpes simplex virus-1 encephalitis in children and impaired responsiveness to poly(I:C) [53].

Localization of TLR receptors is crucial for response to pathogens [4], [54]. We discovered that unique interaction between the ectodomain of TLR3 and UNC93B1 contribute to the receptor trafficking to the cell surface in contrast to TLR9. TLR3-UNC93B1 interaction could control the traffic of TLR3 to compartments where it can be differentially glycosylated [20]. However, the functional role of cell surface TLR3 in mediating immune response to viral infections still needs to be resolved.

## Supporting Information

Figure S1 **Mouse Unc93b1 does not localize on the plasma membrane.** (A) HEK293T cells were transfected with Unc93b1-mCherry-Myc (magenta) and TLR3-mCer (cyan). Plasma membrane was dyed with CTB Alexa 647 (red). Membrane localization was evaluated from plots of normalized fluorescence intensities of Unc93b1-mCherry-Myc and TLR3-mCer and plasma membrane (PM) within 3 μm line profiles (n = 9). Three representative lines are marked on merged images. Images are selected from three independent experiments. Scale bars, 10 μm. (C–D) HEK293T cells were transiently transfected with plasmid encoding TLR3 alone (900 ng DNA/well) or with UNC93B1-mCherry-Myc (C) or Unc93b1-mCherry-Myc (D) (both 10 and 30 ng DNA/well). TLR3 was detected on a Western blot using anti-TLR3 antibody. * indicates the differentially glycosylated form of TLR3. Lower panel shows loading control (non-specific band). The representative data from two experiments are shown. (E) HEK293 cells were transiently transfected with TLR3 (20 ng DNA/well) alone or with UNC93B1-mCherry-Myc/Unc93b1-mCherry-Myc (1 ng DNA/well). Cells were transfected with NF-κB promoter reporter plasmids and *Renilla* reporter plasmid. After 18 h of stimulation with poly(I:C) (10 μg/ml) luciferase activity (RLU) was measured in the cell lysates. The results are represented by mean values with SD from triplicate wells. The representative data from three experiments are shown. Statistical significance is indicated by **, p≤0.05.
(TIF)
