# Supplementary figures and images for: The Ectodomain of TLR3 Receptor Is Required for Its Plasma Membrane Translocation
Source: PLoS One. 2014 Mar 20;9(3):e92391. doi: 10.1371/journal.pone.0092391 (PMC3961339; doi:10.1371/journal.pone.0092391)

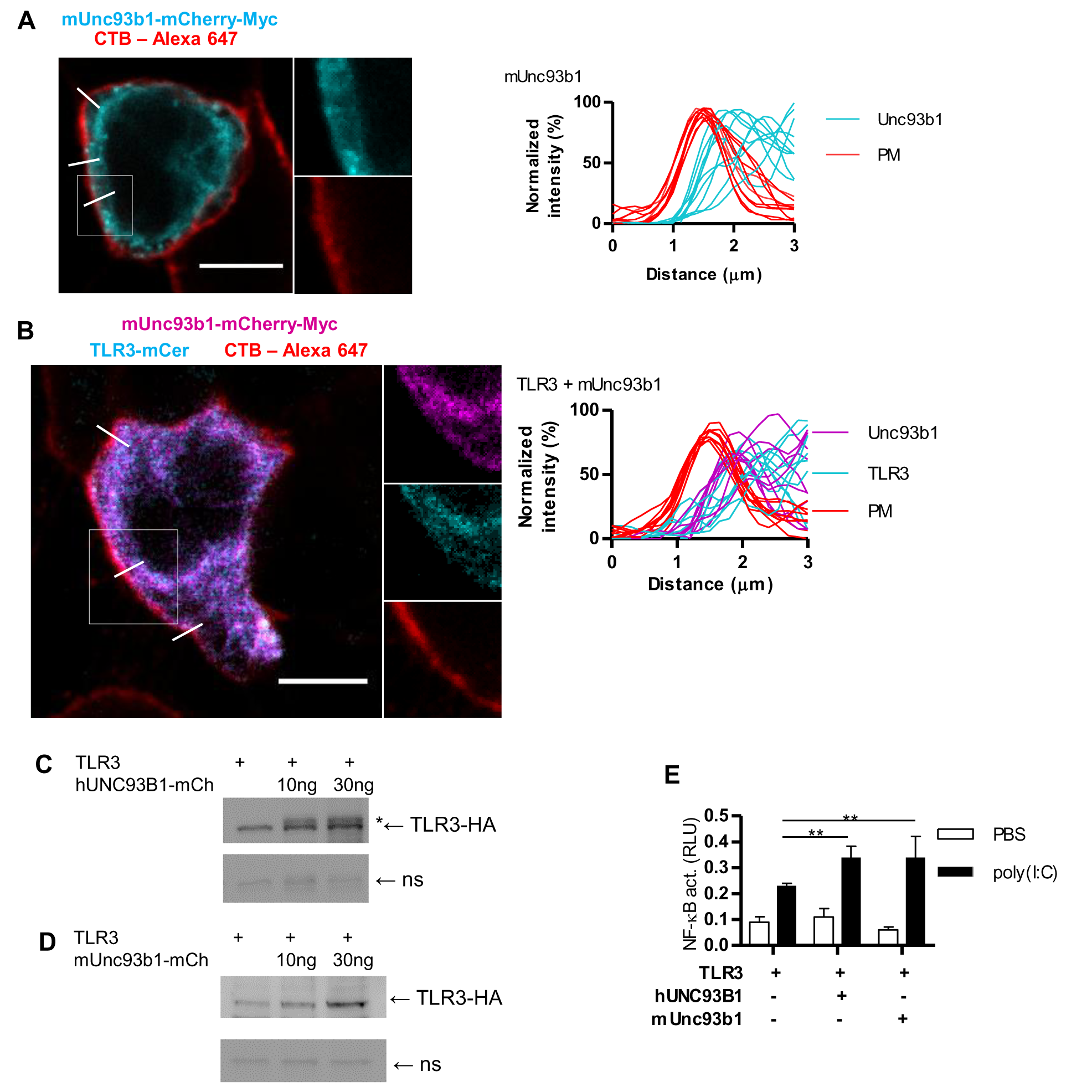

Supplement: Figure S1 — Mouse Unc93b1 does not localize on the plasma membrane. (A) HEK293T cells were transfected with Unc93b1-mCherry-Myc (magenta) and TLR3-mCer (cyan). Plasma membrane was dyed with CTB Alexa 647 (red). Membrane localization was evaluated from plots of normalized fluorescence intensities of Unc93b1-mCherry-Myc and TLR3-mCer and plasma membrane (PM) within 3 μm line profiles (n = 9). Three representative lines are marked on merged images. Images are selected from three independent experiments. Scale bars, 10 μm. (C–D) HEK293T cells were transiently transfected with plasmid encoding TLR3 alone (900 ng DNA/well) or with UNC93B1-mCherry-Myc (C) or Unc93b1-mCherry-Myc (D) (both 10 and 30 ng DNA/well). TLR3 was detected on a Western blot using anti-TLR3 antibody. * indicates the differentially glycosylated form of TLR3. Lower panel shows loading control (non-specific band). The representative data from two experiments are shown. (E) HEK293 cells were transiently transfected with TLR3 (20 ng DNA/well) alone or with UNC93B1-mCherry-Myc/Unc93b1-mCherry-Myc (1 ng DNA/well). Cells were transfected with NF-κB promoter reporter plasmids and Renilla reporter plasmid. After 18 h of stimulation with poly(I:C) (10 μg/ml) luciferase activity (RLU) was measured in the cell lysates. The results are represented by mean values with SD from triplicate wells. The representative data from three experiments are shown. Statistical significance is indicated by **, p≤0.05. (TIF) [file pone.0092391.s001.tif]
